# Correction: Kelebek et al. Exploring the Impact of Infusion Parameters and In Vitro Digestion on the Phenolic Profile and Antioxidant Capacity of Guayusa (*Ilex guayusa* Loes.) Tea Using Liquid Chromatography, Diode Array Detection, and Electrospray Ionization Tandem Mass Spectrometry. *Foods* 2024, *13*, 694

**DOI:** 10.3390/foods13071053

**Published:** 2024-03-29

**Authors:** Hasim Kelebek, Hatice Kubra Sasmaz, Ozge Aksay, Serkan Selli, Ozan Kahraman, Christine Fields

**Affiliations:** 1 Department of Food Engineering, Faculty of Engineering, Adana Alparslan Turkes Science and Technology University, 01250 Adana, Turkey; hkelebek@atu.edu.tr (H.K.); haticemedine95@hotmail.com (H.K.S.); ozgegurler2@gmail.com (O.A.); 2 Department of Food Engineering, Faculty of Engineering, University of Cukurova, 01330 Adana, Turkey; sselli@cu.edu.tr; 3 Applied Food Sciences Inc., 675-B Town Creek Road, Kerrville, TX 78028, USA; cfields@appliedfoods.com

In the original publication [1], there was a mistake in Figure 8 as published. The wrong figure was replaced after enhancing the resolution. Corrected Figure 8 appears below. The authors state that the scientific conclusions are unaffected. This correction was approved by the Academic Editor. The original publication has also been updated.

**Figure 8:**
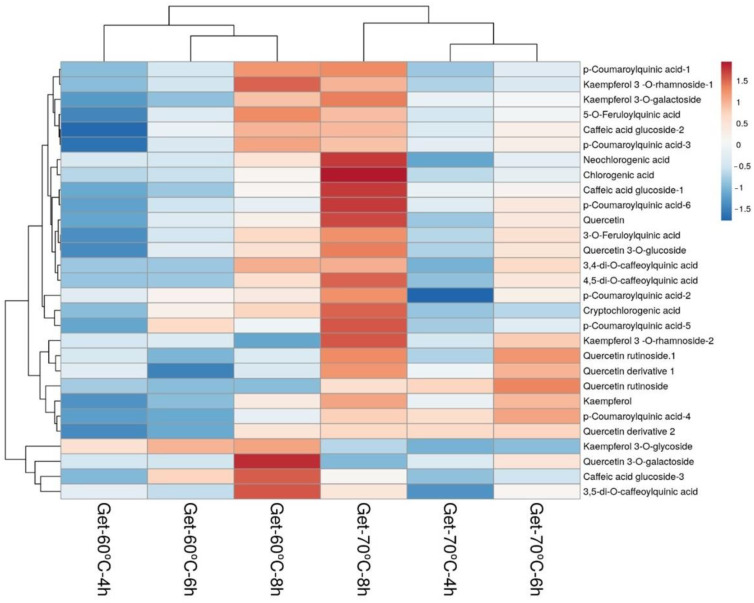
Heatmap of phenolic compounds in Guayusa ethanol–water (Get) infusions. Rows are centered; unit variance scaling is applied to rows. Both rows (29 rows; phenolics) and columns (6 columns; infusions) are clustered using correlation distance and average linkage.
